# Turning over New Leaves

**Published:** 1889-05-18

**Authors:** 


### Turning Over New Leaves.

(Books for Review should be sent to The Editor, The Lodge, Porchester Square, W.)

### BOARD SCHOOL LARYNGITIS.*

A good deal has been said and written about the dangers modern education may bring to the pupils; Dr. Greville Macdonald points out some of the risks run by teachers. A large number of teachers, especially female ones, have applied to him for relief of a form of laryngitis which seems to be different in many respects from that to which other professional voice-users are liable. So characteristic are its symptoms, that Dr. Macdonald says he can often tell that the patient is a teacher simply by the aspect of her throat. The disease itself is an aggravated form of chronic congestion of mucous membrane. Some of the causes that develop it will be obvious to anyone who has visited an elementary school. On opening the door the visitor is simultaneously conscious of two disagreeables: a foul atmosphere and a discordant mingling of many voices. In a room built for about half the number—if air-space be calculated, and not mere standing-room—there are from sixty to 120 children, drawn chiefly from the masses of the "great unwashed." These are divided into two or more classes, of which one may be reciting aloud while the other is writing to dictation. What can the poor teacher of the dictation class do but try to shout down all the little shouters at the other end of the room? "Add to this," says Dr. Macdonald, "the ceaseless mental strain and worry in temper experienced by every class of school-teacher, and especially by those employed in the Board school, to whom there remain none of the usual facilities for correcting insubordination, and we can readily account for the anæmia so prevalent among them."

Another cause is overwork, to which must be added erroneous voice-production. The Board school teacher is not taught to speak, and, perhaps, the best cure for Board school laryngitis—better than the cautery or snipping at the tonsils—would be a sound training in the rules of voice-production, enunciation, and elocution. Actors who have daily to speak in large theatres, and to use a variety of modulation unknown in any other profession, have not yet developed a disease of their own; but we have "clergyman's sore throat" and "Board school laryngitis" among other people who have to use their voices much. The fact is that the latter do not know how to breathe, how to produce the voice, nor how to give clear enunciation to their words. They shout and scream for want of knowledge of any better way of making themselves heard. Unfortunately, moreover, a cold or a sore throat is not considered a sufficient reason for a teacher stopping work. They go on teaching and shouting to their pupils with congested vocal chords and inflamed tonsils; and thereby increase the disease. If they seek help from a doctor, it is usually when the disease has reached a chronic state; and if he prescribes rest as an essential part of the cure, they are apt to go away, scornful of his powers, and ready to pin their faith on the wonder-working lozenges of the chemist at the corner, which relieve the dryness or pain in the throat for perhaps a minute and a half.

But, indeed, this is a disease for which doctors can do little. The ounce of prevention is the thing to be sought, and it can be given only by architects, training-colleges, and school boards themselves. If the acoustics of school-rooms were as carefully studied as those of theatres and concert-halls; if a sound and thorough course of elocution were included among the teacher's studies, and if only one class of moderate size—say, fifty pupils at the most—were carried on simultaneously in the same room, Board school laryngitis would be as rare here as in America, where these things are enforced.

* "Board School Laryngitis." By Greville Macdonald, M.D.Lond., Physician to the Throat Hospital, Golden-square, W. London: Alexander P. Watt, 2, Paternoster-square. Price 1s.

